# Assessment of the Clinical Effectiveness of DRL Orthokeratology Lenses vs. Single-Vision Spectacles in Controlling the Progression of Myopia in Children and Teenagers: 2 Year Retrospective Study

**DOI:** 10.3390/children10020402

**Published:** 2023-02-18

**Authors:** António Queirós, Pauline Beaujeux, Louisette Bloise, Aurélia Chaume, Jean Philippe Colliot, Dominique Plaisant Proust, Perrine Rossi, Bertrand Tritsch, Dominique Bastien Crinon, Jaume Pauné

**Affiliations:** 1 Clinical and Experimental Optometry Research Lab (CEORLab), School of Science University of Minho, 4710-057 Braga, Portugal; 2 Physics Center of Minho and Porto Universities (CF-UM-UP), 4710-057 Braga, Portugal; 3 Clinique 27 Rue de Fleurus, 75006 Paris, France; 4 Espace Médical Cap 3000, Avenue Donadeï, 06700 Saint Laurent du Var, France; 5 Centre Médical Voltaire, 24-26 Rue de Mareville, 54520 Laxou, France; 6 Centre Médical d’Ophtalmologie de Chantilly (SCM), 7 Avenue de Bourbon, 60500 Chantilly, France; 7 Union des Ophtalmologistes du Sud Est, 30 Rue des Electriciens, 13012 Marseille, France; 8 Clinique Saint Hilaire, 2 Place Saint Hilaire, 76000 Rouen, France; 9 Tritsch Bertrand, Espace Ville de France 3 Quai de la Sinn, 68000 Colmar, France; 10 Centre Point Vision, 58 Rue du 14 Juillet, 62300 Lens, France; 11 Teknon Medical Center, 08022 Barcelona, Spain; 12 Faculty of Optics and Optometry Polytechnic, University of Catalonia, 08222 Terrassa, Spain; 13 Optometry School Optometry, University of Montreal, Montreal, QC H3T 1J4, Canada

**Keywords:** orthokeratology, refraction, control of myopia progression, DRL lenses

## Abstract

The purpose of this study was to assess the effect of orthokeratology treatment with DRL lenses on the control of myopia progression compared with single vision glasses users (monofocal glasses). It was also possible to analyze the clinical efficacy of orthokeratology treatment with DRL lenses for myopia correction in children and adolescents in a 2 year retrospective, multicenter study, performed in eight different ophthalmology centers in France. A total of 360 data records of children and adolescents with myopia between −0.50 D and −7.00 D at baseline visit, who completed treatment and had a centered outcome, were selected for the study from a database of 1271. The final sample included subjects undergoing orthokeratology treatment with DRL lenses (*n* = 211 eyes) and spectacle wearers (*n* = 149 eyes). After one year of treatment, the data analysis shows that the DRL lens has a refractive myopia progression control rate of 78.5% compared with the spectacle wearers (DRL M change = −0.10 ± 0.25 D, *p* < 0.001 Wilcoxon test and Glasses M change = −0.44 ± 0.38 D, *p* < 0.001 Wilcoxon test). Similar results were found after 2 years of treatment (80% with 310 eyes). This study showed the clinical efficacy of orthokeratology DRL lenses compared to monofocal spectacle wearers in controlling myopia progression in children and adolescents in a 2 year retrospective study.

## 1. Introduction

The prevalence of myopia has grown significantly in the last decades, affecting mainly Asian countries; currently it is a global concern affecting North America and Europe. Recent estimates suggest that half of the world’s population will become myopic by 2050, with problems associated with increased risk of different eye pathologies, some of them affecting vision permanently, with future indications of blindness. Thus, the prevalence of myopia is expected to increase to 55% in Europe, 60% in North America, and 65% in Asia [1,2]. The prevalence of myopia is increasing in Europe. Recent publications showed the age-standardized myopia prevalence for those completing primary, secondary, and higher education was 25.4%, 29.1%, and 36.6%, respectively [3,4,5,6,7]. In France, a study of over 100,429 individuals, performed in four different eye treatment centers, showed prevalence of mild, moderate, high, and very high myopia were, respectively, 25.1%, 10.6%, 3.4%, and 0.5% [8]. The overall prevalence was higher in the 20 to 39 year olds groups with a rate of 52.4%. This has significant implications for the future; increasing myopia prevalence, and specifically high levels in younger individuals, will potentially result in an increasing burden of associated visual impairment in the future.

Systematized compensation of refractive error started in the 15th century with the invention of spectacles. Several alternatives to spectacles have been developed since the last century, from rigid contact lenses (CL) more than 60 years ago, through gas permeable CL, hydrophilic and disposable lenses to orthokeratology lenses, a rigid gas-permeable CL with an inverse geometry, in the last 40 years [9,10]. Orthokeratology (OK), a reversible technique, aims at compensating spherical (up to −8.00 D) and regular astigmatic (up to −4.00 D) refractive defects by redistributing the corneal epithelial tissue through the application of OK lenses during sleep, so that the patient can do without any visual compensation during the day [11].

The growing prevalence of myopia worldwide and its associated pathologic complications have raised public concern for identifying effective solutions to control myopia [12]. Orthokeratology (OK), which can be used to temporarily correct refractive errors by wearing specially designed reverse-geometry rigid gas permeable lenses overnight to reshape the cornea, has been considered an effective optical intervention for retarding myopia progression in children [13]. The inhibitory effect on axial elongation in myopic children for 2-year OK treatment has been reported to vary from 32% to 63% [14,15,16,17,18,19,20]. This variation in results may be explained, in part, by the usage of different lens designs and their application in different populations. We consider myopia progression when a corrected myopic eye exhibits variations in its ocular optical components with the end result that the eye focuses parallel light rays coming from infinity before the retina. The progression of myopia has been associated with several risk factors including genetic predisposition, inadequate accommodative response, high AC/A ratio, time on tasks performed in near vision, less outdoor activity, and the hypermetropic blur value of the peripheral retina [21,22].

The onset of myopia is associated with the age of 6–8 years and progresses over the following 10–15 years, stabilizing during the later years of adolescence and early adulthood [23]. Associated with this is the early onset of myopia which allows it to progress over a longer period of time to higher values. Thus, the age at which myopia manifests itself serves as a predictor for the final quantification of myopia, generally associated with high values [23]. This association is related to the development of serious ocular complications such as cataracts, glaucoma, retinal detachment, and maculopathy at older ages due to the growth of the eye and the stresses caused to it [2,24].

The exponential rise in myopia, especially during the COVID-19 pandemic, has been attributed to changes in lifestyle. The efforts to prevent the progression of myopia in the form of spectacles, contact lenses, and orthokeratology have shown some promising results. However, the dimensions of risks and benefits are vast and need continuous scrutiny by researchers and clinicians. Orthokeratology reshapes the corneal surface temporally through a reverse-geometry design by wearing gas permeable contact lenses at night. It flattens the central cornea, making it thinner and redistributing the epithelial cells to the mid-periphery of the cornea. Thus, it is supposed to retard myopia progression by reducing the hyperopic peripheral refractive error, which in turn results in a decrease in AL elongation [25,26,27].

The Retardation of Myopia in Orthokeratology (ROMIO) Study compared AL elongation in children aged 6 to 10 assigned to wear either OK lenses or single vision glasses users over 2 years of follow-up and revealed that OK lenses lead to a slower overall reduction in AL elongation, particularly in younger myopic children [15]. Therefore, larger-scale, randomized trials are required for further evaluation of the real efficacy of OK. Moreover, there is a need to focus on the risk of myopia rebound after discontinuation of OK lenses. Yang et al. revealed in their retrospective study that the corneal morphology and central corneal thickness (CCT) returned to the original values after only 3 months of OK lenses discontinuation; however, no effect of myopia rebound was shown [28]. However, this type of treatment, and specifically when used on children, should always be continuously monitored by parents and eye care professionals.

There is much worldwide research on the effectivity of the orthokeratology in retarding myopia progression, but there are not many publications on European children and none on the French population. The objective of this retrospective study is to assess the normal myopia progression in French children and its prevention using a DRL orthokeratology-specific design.

## 2. Materials and Methods

### 2.1. Subjects

We retrospectively collected data from subjects undergoing orthokeratology treatment with the DRL lens (*n* = 590 eyes with DRL) in 8 ophthalmology clinical centers in France, and in 3 of these centers data from patients wearing glasses were also collected (*n* = 684 eyes with glasses). Only complete patient files that met the study objectives were considered for data collection. Specifically, refraction and visual acuity data, as well as records of reported adverse events (0–4 on the CCLRU scale).

The protocol and study procedures were reviewed and approved by Study, published on the Health Data Hub n°F20220113001828, France. This study followed the principles of the Declaration of Helsinki. All parents of the participants provided informed consent after they received an explanation of the nature, procedures, and consequences of the study.

To facilitate the analysis of refractive error, the vectorial components mentioned by Thibos et al. (M, J0 and J45) were calculated and used for analysis [29]. To standardize the Sph and M variable (M = Sph + Cyl/2) at the beginning of treatment for the DRL and spectacle samples, subjects were randomly selected as described in Table 1 and Table 2. Only patients with good visual acuity and no reports of adverse events before the period of recruitment were taken into account. Thus, from the collected sample of 1274 eyes, those who met the inclusion criteria and were in agreement with the randomization to obtain values no different from Sph and M, made the final sample 360 subjects (DRL lens patients *n* = 211 and glasses control patients *n* = 149).

### 2.2. Measurements

All patients underwent a full ophthalmology service and all inclusion and exclusion criteria were analyzed. Monocular measurements of the subjective noncycloplegic refraction were recorded. The endpoint of refraction was established by the criterion of maximum plus for best visual acuity [27]. The OK and SV groups were divided. The parameters evaluated were refraction (sphere, cylinder, and axis), visual acuity, central visual efficiency (VE = 0.2^(MAR−1)/9^), and index visual security (VA_pos/VA_pre) at baseline, after 1 year, and 2 years from the beginning of the study.

The inclusion criteria required age between 5 and 17 years at baseline visit; patient with a diagnosis of myopia with a subjective refraction between −0.50 D and −7.00 D at baseline visit; patient with a diagnosis of astigmatism between 0 and 4 diopters at baseline visit; and patient with a best-corrected visual acuity (VA) of 10/10 (0.0logMAR) or better in both eyes at baseline visit. For the group “DRL Orthokeratology lenses”, patient who initiates the use of DRL Orthokeratology lenses between 1 January 2016 and 30 June 2020 and who had at least 3 consultation visits: baseline, 1 year +/− 1 months and 2 years +/− 1 months; for the group “Single-vision (SV) spectacles”, patient who initiates or has already used single-vision spectacles between 1 January 2016 and 30 June 2020 and who had at least 3 consultation visits: baseline, 1 year +/− 1 month and 2 years +/− 1 month. All patients with ocular pathologies, who had undergone eye surgery, who were on any prescription medication or with adverse effects on the CCLRU scale were excluded from the analysis in this study. Additionally, patients with amblyopia or strabismus were also excluded.

### 2.3. Statistical Analysis

The statistical package SPSS v.21 (SPSS Inc., Chicago, IL, USA) was used to conduct the statistical analysis. The Kolmogorov–Smirnov Test was applied to evaluate the normality of data distribution. The chi-squared test was used to analyze the variables between the groups. The Mann–Whitney U-test or Kruskal–Wallis test were used for the analysis of differences in refraction variables before and after treatment at one and two years. Comparison between the three visits was made with the repeated measures ANOVA test with Bonferroni adjustment. A *p*-value < 0.05 was accepted as statistically significant.

## 3. Results

### 3.1. Comparison of the Study at 12 Months

The data analysis shows that the orthokeratology treatment with the DRL lens (*n* = 211) in both the spherical component of refraction (Sph) and the vector component of the spherical equivalent (M) shows smaller increments in diopters compared to the subjects who wore glasses (*n* = 149). In fact, in M the value went from −2.95 D to −3.04 D in the subjects with DRL lens and from −2.80 D to −3.24 D in the subjects with glasses (Table 3). Thus, there was a difference of over 0.34 D in the subjects with glasses compared to those with the DRL lens. This analysis shows that the DRL lens has a refractive myopia progression control rate of 79% compared with the spectacle wearers. For the vector components of astigmatism J0 and J45, we observed relatively small clinical increments after 12 months (<0.10 D), although the statistical values found were statistically significant. The data also show a high percentage in visual efficiency and a high safety ratio with both treatments, i.e., the treatments show that visual acuities at the end of the treatments do not worsen significantly in clinical terms.

### 3.2. Comparison of the Study at 24 Months

When considering only the subjects with 24 months follow-up (loss of −14% of subjects), the sample is reduced to subjects with DRL lenses of *n* = 184 and with spectacle wearers of *n* = 126. This loss of 50 subjects was due to the subjects giving up or not attending the follow-up period, which is why they could not be considered in the second phase of the study (24 months follow-up).

As we can see in Table 4, when comparing only the subjects with the two-year treatment (*n* = 310), we found that in terms of the refractive error there were statistically significant differences for Sph and M changes in both groups. Showing an increment in myopia of −0.12 ± 0.32 D and −0.70 ± 0.63 D in Sph terms and −0.15 ± 0.33 D and −0.76 ± 0.33 D in M terms for DRL and SV, respectively. In light of the results, we can conclude that in spectacle wearers, the increment in myopia was −0.58 ± 0.06 D in Sph and −0.62 ± 0.06 D in M terms, more than contact lenses DRL wearers. In the 12 month analysis, we observed clinically insignificant values for the astigmatism components (<0.05 D), despite the statistical significance found.

In order to better understand the evolution of the two treatments, repeated measures ANOVA analysis was performed. Table 5 shows the separate analysis of the subjects in the two treatments at baseline, at one year, and at the end of two years.

The first analysis is that progression with statistical significance is noted in the DRL lens at 12 months (Bonferroni adjustment, V1–V2) of M = −0.10 D, while in the spectacle wearers this value was M = −0.43 D more (Bonferroni adjustment, V1–V2). A more careful analysis of the results after 24 months (in comparison to 12 months of treatment) shows that in the case of DRL treatment, the myopia progression does not change significantly (diff M = −0.04 D more, Bonferroni adjustment) while in spectacle wearers the difference from 12 months to 24 months is an M = −0.33 D increase in myopia with statistical significance (Bonferroni adjustment, V2–V3). Additionally, in the longitudinal analysis of the sample in relation to J0 and J45, statistically significant differences were found with the exception of J45 for glasses, being most evident between visit 1 (baseline) and visit 2 (6 months).

**Figure 1:**
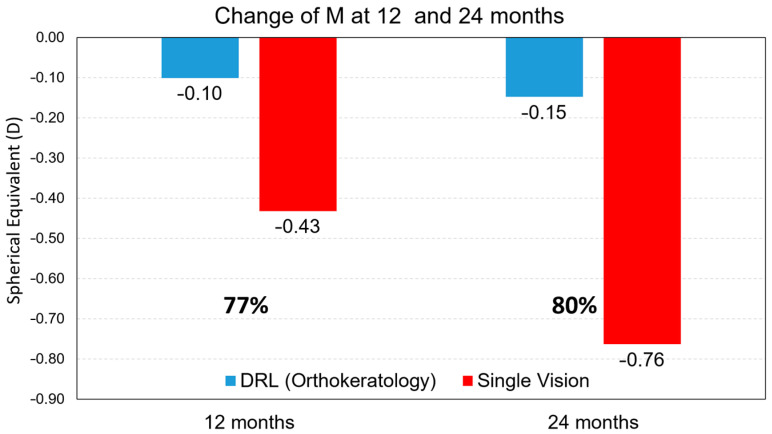
Myopia progression retention rate values, for the M component, after 12 months and after 24 months of treatment between treatments.

The analysis of myopia progression control shows that the DRL lens shows rates of 77% in the first year and 80% in the second year compared to the spectacle-wearing subjects (Figure 1).

### 3.3. Comparison of the Study at 24 Months for the M Component as a Function of Age of the Study at 24 Months

**Figure 2:**
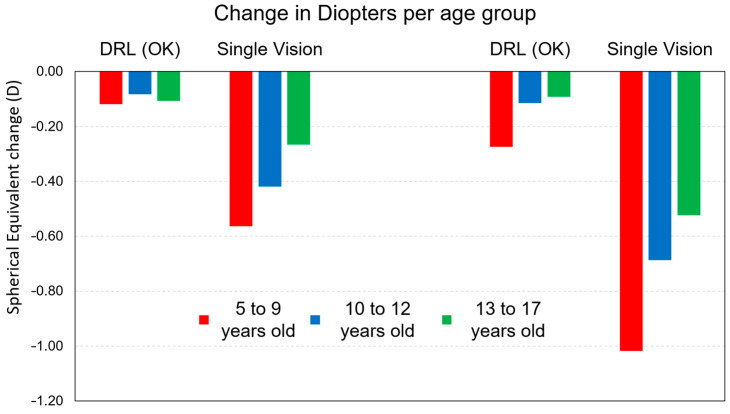
Myopia progression retention rate values for the M component after 12 months and after 24 months of treatment between treatments.

The detailed analysis according to age groups shows that in all the groups analyzed there was an increase in refraction M being greater in the youngest subjects for both treatments (Figure 2). However, we found that this increase was much greater in glasses than in CL DRL (Table 6). Significant effects on the control of myopia progression with DRL contact lenses were found in the three age groups, respectively, M < 0.74 D with 73% for the age group 5–9 years, M < 0.58 D with 84% for the age group 10–12 years, and M < 0.43 D with 83% for the age group 13–17 years.

The analysis of Figure 2 and the results in Table 7 show that there is indeed an increase in myopia in both groups. However, we found that the differences for the DRL treatment are significantly smaller than for glasses in the three age groups. In Table 7, despite the statistical difference between visits with any treatment, the DRL lenses show that between visit 2 and visit 3 this difference is no longer significant (*p* > 0.05, Repeated measures, Bonferroni adjustment). That is, while with glasses the M component continues to evolve after one year and after two years in the three age groups, in CL DRL users after one year the evolution is no longer statistically significant for the second year and there is thus an efficiency in the control of myopia progression (Bonferroni only for Visit_1 with Visit_2 (V1–V2) and Visit_1 with Visit_3 (V1–V3) and not Visit_2 with Visit_3 to DRL).

**Figure 3:**
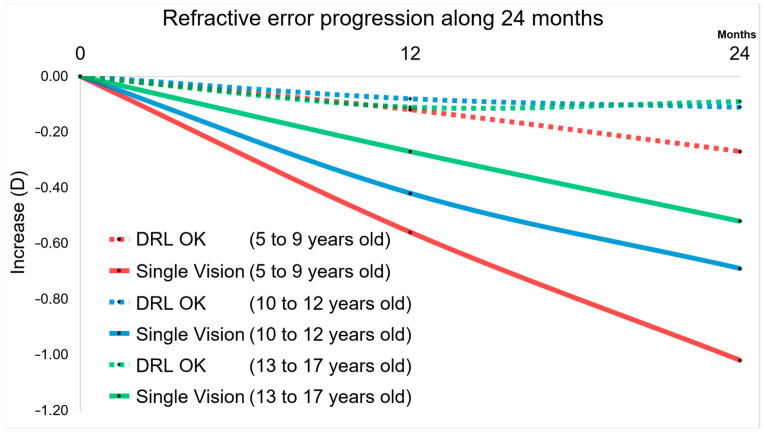
Plot with the M component for all groups over 24 months. DRL and Single Vision Glasses for 5–9, 10–12, and 13–17 years old. Ratios of Refractive error changes are evident for SVG and a sign of slowing is appreciated for higher age groups, showing that myopia is not stopped at the age of 17 years.

These results can be more easily observed in the graphical analysis of Figure 3. In fact, both at 12 months and 24 months, it is visible the impact of DRL lenses in a smaller increment compared to glasses (SVG). This result is much more expressive in the 5–9 years age group and in the 10–12 years age group.

**Figure 4:**
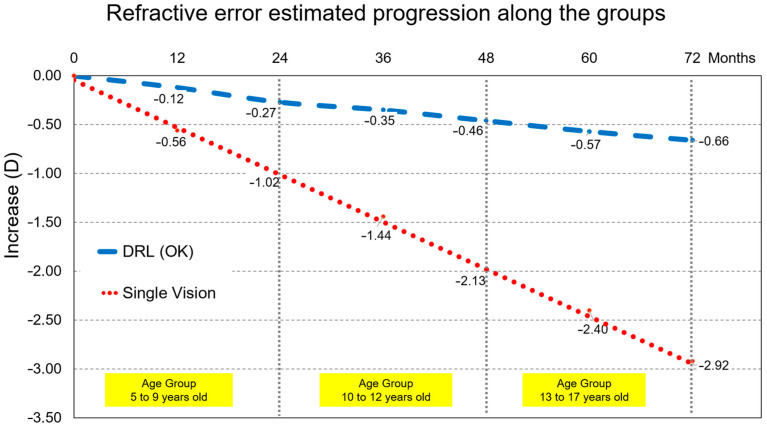
Myopia progression for all participants linking the end and the beginning of each age group. Even not being same individuals, an overall trend may be inferred for both groups. Thus, a projected different M component change by −2.26 D after 6 years for a child starting myopia at 8 years compared to wearing orthokeratology lenses or single vision lenses. This accounts for a 3-fold increase risk in macular degeneration or retinal detachment.

On the other hand, extrapolating these results across age groups we can see (Figure 4) that the efficiency of treatment is fruitful and efficient across age and growth of children/youth.

## 4. Discussion

The study and development of optical devices for the correction of refractive defects is important not only for refractive compensation but also because it influences the quality of life of the subjects [30]. Added to this importance is a greater one that concerns the evolution of refractive errors. Specifically myopia, which is associated with an excessive elongation of the eyeball resulting in blurred far vision. This phenomenon, which although partly genetic, has mainly environmental risk factors, such as low exposure to sunlight [31] or an extended close reading [12,32]. Thus, today we are faced with generations that are more nearsighted than previous generations, and increasingly at a younger age. If detected early, managing myopia control can help delay its progression in children and ensure a better quality of life [33]. The control of myopic progression has been accomplished either with medications or with optical devices. Longitudinal studies to study this effectiveness show 50–70% lower myopia increase in subjects wearing orthokeratology contact lenses [12,16,34]. Added to this evidence is the fact that the treatment is safe, reversible, and has no rebound effect [28,35]. It is commonly thought that the occurrence and progression of myopia are caused by the peripheral hyperopic defocus [36]. In animal models and in humans, the progression of myopia is influenced by the visual input at the retina [37]. Many studies have shown that increasing the peripheral myopic defocus slows the progression of myopia [25,38,39]. The results found by many authors are consistent with our results. The difference found in our study for the spherical equivalent between DRL lens treatment and glasses was M = −0.35 D (*p* < 0.001 Mann–Whitney U-test, Table 3) less in the first year and M = −0.60 D (*p* < 0.001 Mann–Whitney U-test, Table 4) less in the second year of treatment for the DRL lens wearers. This is in agreement with the studies that show us that a decrease of 0.30 D SER per year plays an important role in the long-term control of myopia [14,40,41].

This report describes the clinical study conducted in eight ophthalmology clinics in France during the years 2016 to 2020. This study was based on the clinical data of children and adolescents between 5 and 17 years old. In a retrospective way, we analyzed files of patients who used glasses (Single-vision—SV) and orthokeratology contact lenses (DRL Orthokeratology) with a consultation record of at least 1 or 2 years performed by the same ophthalmologist during that period. After data collection, refraction variables were studied and their evolution over two years was analyzed. A total of 360 clinical files were studied, of which 211 were orthokeratology contact lens wearers and 149 were spectacle wearers, which in this study served as the control group. The clinical refraction of both groups is described in Table 2.

In the last two decades, there have been several longitudinal studies with orthokeratology contact lenses. Known by the orthokeratology technique for night use, they are associated with a decrease in central refraction and an increase in corneal power in the periphery, making the peripheral blur initially hypermetropic become myopic. From the first study with Pauline Cho until today, the values found vary from 30 to 60% when compared to other types of contact lenses or even glasses [15,19,34,42,43,44]. Various treatment effects have been reported for OK depending on age and initial myopia value. In the myopia control study with orthokeratology, the effectiveness of OK in controlling myopia was found to be better in younger children than in older subjects, as found in our study [15].

Recently, two published studies, where different types of contact lenses were compared in the Chinese population at similar ages, show similar percentage values in the retention of myopia progression with DRL OK lenses in the range of 76 to 85% [45,46]. Although our results are similar to those found with the same lens type and age, they show a high control on myopia progression in a Caucasian population. This analysis performed by different age groups also shows extraordinary results. Thus, by analyzing Table 8 we can observe the importance of controlling myopia progression at younger ages. Several questions arise when changing from habitual treatments to correct myopia to treatments that pretend to modify its progression. One of the most important questions for the clinicians is “when to start the treatment”. To answer this question, at least three factors need to be considered: age of the patient, refractive error, and the potential of progression [47]. Currently, several factors are identified as predictors of faster and longer myopia progression, which may lead to higher amounts of myopia in adulthood [48], and consequently, an increase in the risk of ocular pathologies associated with myopia, including retinal detachment, maculopathy, glaucoma, or cataracts [49]. Early onset of myopia has been associated with greater and faster progression. The same has been found when both biological parents are myopic compared to children with only one or no myopic parent [50]. The educational level and the rate of near-work and outdoor activities have also been related to a higher risk of myopia. Low levels of outdoor activity seem to result in a higher risk of myopia, particularly when associated with high levels of near-work. This might be related to the later association between higher educational levels and myopia [51]. These are topics that need to be considered as potential increased risks of myopia, which may benefit from early intervention to minimize the long-term effects of myopia progression. Apparently, younger myopic children tend to experience faster axial elongation and may benefit from early treatment [52]. Therefore, early initiation of the treatment may be necessary to reduce the prevalence of high myopia or elongation [53]. Thorn et al. suggested three models for the progression and showed that its potential differs with the age of onset [54]. More importantly, these models define the “window of opportunity to treat”. Considering that myopia control treatments intend to slow the progression rate, it is necessary to step in and initiate treatment during the fastest progression phase, as this would potentially maximize the final benefit to achieving lower axial growth in adulthood.

The results of our study truly show this. Thus, the analysis of Figure 3 and Figure 4 describe the so-called window of opportunity to intervene as early as possible to control myopia progression. Optical treatments, such as orthokeratology (DRL in this study), show their effectiveness in arresting myopia progression, both at 12 months of the study and at 24 months, where a smaller increase in refraction was observed compared to glasses that is more evident at younger ages. The importance of this retention will be more evident given the effect of greater axial elongation associated with high myopia (≤−5.00 D) that leads to structural changes in the posterior segment of the eye (including posterior staphyloma, myopic maculopathy, and optic neuropathy associated with high myopia) and can lead to considerable visual acuity loss. Thus, by being able to control the evolution of refraction in myopic children, we will be controlling the consequences of visual impairment at later ages [55,56]. Failure to treat these subjects early will cause subjects with worse myopia to incur the costs of specialized eye care, or even specialized optical aids, in the order of a few billion dollars per year; associated with this is a reduced quality of life due to adverse influences of psychological, aesthetic, functional, and financial factors [57]. Finally, the fact should be highlighted that in two-year longitudinal terms, the DRL treatment showed in this study to be efficient and safe in refractive terms and in terms of visual acuity with difference values of less than ±1%.

Although the retrospective collection in this study does not address the axial length of the eye, because many eye centers do not have the equipment or it was not considered as a consultation protocol, it is clear that there is a direct relationship and high correlation between axial length and refraction of myopic eyes [58,59]. Therefore, any efforts that can be made in the future to keep myopic eyes below −3.00 D of refraction may help to avoid increases in the prevalence of visual impairment in the world and may be associated with maintaining a higher rate of working-age population [60]. This analysis may also be associated with programs to prevent and monitor myopic refractive enhancement in children that will allow clinicians to monitor eye growth in children. Therefore, early detection of eye overgrowth may facilitate decision-making regarding interventions to prevent and/or control myopia [61]. Another recent paper showed through a logistic regression that each additional year of age and each additional diopter of initial myopia decreased the probability of faster myopia progression (0.10 mm/year), where OR = 1.23; 2.19 [IC 95%] and OR = 1.08; 3.47 [IC 95%], respectively. Orthokeratology treatment decreased the probability of myopia progression greater than 0.10 mm/year by 8–23%. Axial length did not change significantly in children over 11 years of age or in children with myopia greater than −4.00 D with orthokeratology treatment [52]. Thus, this study reinforces the importance of starting orthokeratology treatment in myopic eyes in children at the earliest possible age in the Caucasian population.

## 5. Conclusions

In conclusion, all efforts must be directed towards slowing the progression of myopia. The progression of myopia follows a sigmoidal curve, with a slower start and end and an intermediate phase of faster growth where it is important to intervene. The progression of myopia has several patterns that are conditioned by factors such as the age of onset and the initial value at which they start, it tends to slow down its rate of progression around 16–17 years of age. Regarding the use of effective optical devices, the risk–benefit ratio combined with more information for patients and parents are important going forward. Thus, the socio-economic outcomes that the myopia pandemic may bring in the future can be minimized at the public health level to win this fight against myopia progression.

## Figures and Tables

**Table 1:** Demographics of the qualitative variables at the beginning of treatment.

		DRL (*n* = 211)	Glasses (*n* = 149)	Total	*p* *
Gender	Female	116	76	192	0.457
	Male	95	73	168	
Age group	5–9 years	80	73	153	0.055
	10–12 years	98	65	163	
	13–17 years	33	11	44	

* Pearson’s chi-square.

**Table 2:** Demographic data of quantitative variables at the beginning of treatment (*n* = 360).

	DRL	Glasses	Difference	*p* *
Age (years)	11.51 ± 2.66	10.74 ± 2.57	0.77 ± 0.28	0.067
Sph (D)	−2.61 ± 1.27	−2.49 ± 1.20	−0.11 ± 0.13	0.227
Cyl (D)	−0.67 ± 0.69	−0.60 ± 0.70	−0.07 ± 0.07	0.127
M (D)	−2.95 ± 1.35	−2.80 ± 1.34	−0.15 ± 0.14	0.111
J0 (D)	0.19 ± 0.40	0.17 ± 0.36	0.02 ± 0.04	0.909
J45 (D)	−0.01 ± 0.18	0.00 ± 0.23	−0.01 ± 0.02	0.753
VA (LogMAR)	0.00 ± 0.01	0.00 ± 0.02	0.00 ± 0.00	0.100

* Mann–Whitney U-test.

**Table 3:** Data from the longitudinal study at 12 months of treatment in the comparison between DRL and glasses (*n* = 360).

		Baseline	12 Months	Difference	*p* (a)
Sph (D)	DRL	−2.61 ± 1.27	−2.67 ± 1.30	−0.06 ± 0.24	<0.001
	Glasses	−2.49 ± 1.20	−2.90 ± 1.23	−0.41 ± 0.38	<0.001
	*p* (b)	0.227	0.107	<0.001	
Cyl (D)	DRL	−0.67 ± 0.69	−0.75 ± 0.69	−0.07 ± 0.14	<0.001
	Glasses	−0.60 ± 0.70	−0.67 ± 0.72	−0.08 ± 0.14	<0.001
	*p* (b)	0.127	0.051	0.251	
M (D)	DRL	−2.95 ± 1.35	−3.04 ± 1.39	−0.10 ± 0.25	<0.001
	Glasses	−2.80 ± 1.34	−3.24 ± 1.35	−0.44 ± 0.38	<0.001
	*p* (b)	0.111	0.222	<0.001	
J0 (D)	DRL	0.19 ± 0.40	0.12 ± 0.42	−0.08 ± 0.36	0.074
	Glasses	0.17 ± 0.36	0.20 ± 0.38	0.03 ± 0.07	<0.001
	*p* (b)	0.909	0.045	<0.001	
J45 (D)	DRL	−0.01 ± 0.18	0.03 ± 0.26	0.04 ± 0.18	0.001
	Glasses	0.00 ± 0.23	0.01 ± 0.25	0.00 ± 0.06	0.295
	*p* (b)	0.753	0.484	0.497	

(a) Wilcoxon test (b) Mann–Whitney U-test.

**Table 4:** Data from the longitudinal study at 24 months of treatment in the comparison between DRL and glasses (*n* = 310).

		Baseline	24 Months	Difference	*p* (a)
Sph (D)	DRL	−2.70 ± 1.31	−2.82 ± 1.36	−0.12 ± 0.32	<0.001
	Glasses	−2.50 ± 1.14	−3.20 ± 1.35	−0.70 ± 0.63	<0.001
	*p* (b)	0.132	0.017	<0.001	
Cyl (D)	DRL	−0.69 ± 0.70	−0.75 ± 0.68	−0.06 ± 0.13	<0.001
	Glasses	−0.55 ± 0.65	−0.67 ± 0.70	−0.12 ± 0.21	<0.001
	*p* (b)	0.036	0.118	0.083	
M (D)	DRL	−3.05 ± 1.39	−3.19 ± 1.44	−0.15 ± 0.33	<0.001
	Glasses	−2.78 ± 1.26	−3.54 ± 1.44	−0.76 ± 0.63	<0.001
	*p* (b)	0.058	0.048	<0.001	
J0 (D)	DRL	0.20 ± 0.41	0.15 ± 0.40	0.05 ± 0.36	0.377
	Glasses	0.15 ± 0.36	0.21 ± 0.39	−0.06 ± 0.11	<0.001
	*p* (b)	0.396	0.225	<0.001	
J45 (D)	DRL	0.00 ± 0.18	0.03 ± 0.27	−0.02 ± 0.19	0.021
	Glasses	0.01 ± 0.17	0.02 ± 0.19	−0.01 ± 0.07	0.146
	*p* (b)	0.789	0.688	0.758	

(a) Wilcoxon test (b) Mann–Whitney U-test.

**Table 5:** Data from the longitudinal study at 12 and 24 months of treatment in the comparison between DRL and glasses. Inter-visit analysis with Bonferroni adjustment test presented.

		Visit 1 (V1) Baseline	Visit 2 (V2) 12 Months	Visit 3 (V3) 24 Months	*p* *	*p* **
Sph (D)	DRL	−2.70 ± 1.31	−2.76 ± 1.33	−2.82 ± 1.36	<0.001	V1–V2,V1–V3
	Glasses	−2.50 ± 1.14	−2.90 ± 1.21	−3.20 ± 1.35	<0.001	V1–V2,V1–V3,V2–V3
Cyl (D)	DRL	−0.69 ± 0.70	−0.77 ± 0.70	−0.75 ± 0.68	<0.001	V1–V2,V1–V3
	Glasses	−0.55 ± 0.65	−0.62 ± 0.68	−0.67 ± 0.70	<0.001	V1–V2,V1–V3,V2–V3
M (D)	DRL	−3.05 ± 1.39	−3.15 ± 1.42	−3.19 ± 1.44	<0.001	V1–V2,V1–V3
	Glasses	−2.78 ± 1.26	−3.21 ± 1.30	−3.54 ± 1.44	<0.001	V1–V2,V1–V3,V2–V3
J0 (D)	DRL	0.20 ± 0.41	0.12 ± 0.43	0.15 ± 0.40	0.011	V1–V2
	Glasses	0.15 ± 0.36	0.18 ± 0.38	0.21 ± 0.39	<0.001	V1–V2,V1–V3,V2–V3
J45 (D)	DRL	0.00 ± 0.18	0.05 ± 0.27	0.03 ± 0.27	0.001	V1–V2
	Glasses	0.01 ± 0.17	0.01 ± 0.18	0.02 ± 0.19	0.416	---

(*) Repeated measures; (**) Bonferroni adjustment; V1—visit 1 baseline; V2—visit 2 at 12 months; V3—visit 3 at 24 months.

**Table 6:** Data from the longitudinal study at 24 months of treatment in the comparison between DRL and glasses for the M component of refraction.

M (D)	*n*	Age Group	Visit 1 Baseline	Visit 3 24 Months	Difference	*p* (a)
DRL	47	5–9	−3.14 ± 1.23	−3.42 ± 1.40	−0.27 ± 0.50	<0.001
	74	10–12	−2.93 ± 1.46	−3.05 ± 1.45	−0.11 ± 0.24	<0.001
	63	13–17	−3.11 ± 1.43	−3.20 ± 1.44	−0.09 ± 0.24	0.002
Glasses	45	5–9	−2.38 ± 1.10	−3.40 ± 1.40	−1.01 ± 0.75	<0.001
	49	10–12	−2.66 ± 1.15	−3.34 ± 1.36	−0.69 ± 0.51	<0.001
	32	13–17	−3.53 ± 1.33	−4.05 ± 1.54	−0.52 ± 0.46	<0.001

(a) Wilcoxon test.

**Table 7:** Data from the longitudinal study at 1 year of treatment in the comparison between DRL and glasses for the M component of refraction.

M (D)	*n*	Age Group	Visit 1 (V1) Baseline	Visit 2 (V2) 12 Months	Visit 3 (V3) 24 Months	*p* *	*p* **
DRL	47	5–9	−3.14 ± 1.23	−3.26 ± 1.27	−3.42 ± 1.40	<0.001	V1–V2,V1–V3
	74	10–12	−2.93 ± 1.46	−3.02 ± 1.46	−3.05 ± 1.45	<0.001	V1–V2,V1–V3
	63	13–17	−3.11 ± 1.43	−3.22 ± 1.49	−3.20 ± 1.44	0.009	V1–V2,V1–V3
Glasses	45	5–9	−2.38 ± 1.10	−2.95 ± 1.21	−3.40 ± 1.40	<0.001	V1–V2,V1–V3,V2–V3
	49	10–12	−2.66 ± 1.15	−3.08 ± 1.25	−3.34 ± 1.36	<0.001	V1–V2,V1–V3,V2–V3
	32	13–17	−3.53 ± 1.33	−3.79 ± 1.35	−4.05 ± 1.54	<0.001	V1–V2,V1–V3,V2–V3

(*) Repeated measures (**) Bonferroni adjustment test.

**Table 8:** Difference values between DRL and glasses treatments as a function of subjects’ age group for the M component of refraction (Comparison at one year and two years from the beginning of treatment).

Age Group (years)	Diff DRL (D) V2–V1	Diff Glasses (D) V2–V1	% Reduction	Diff DRL (D) V3–V1	Diff Glasses (D) V3–V1	% Reduction
5–9	−0.12	−0.56	−78.87	−0.27	−1.02	−73.08
10–12	−0.08	−0.42	−80.14	−0.11	−0.69	−83.26
13–17	−0.11	−0.27	−59.72	−0.09	−0.52	−82.50

## Data Availability

The materials that support this manuscript are available from the corresponding author upon reasonable request.
